# Dr. Stephan Harbarth reflects on the success of his “hospital diplomat” approach to epidemiology

**DOI:** 10.1017/ash.2025.10256

**Published:** 2025-12-26

**Authors:** Stephan Jurgen Harbarth

**Affiliations:** Geneva University Hospitals and Faculty of Medicine, Geneva, Switzerland

## Abstract

Professor Stephan Harbarth obtained his medical degree from the University of Munich in 1993. He completed postgraduate training in internal medicine and infectious diseases in both Munich and Geneva. After serving as a clinical research fellow in infectious diseases at Geneva University Hospitals (HUG), he pursued postgraduate studies in epidemiology at Harvard School of Public Health, earning a Master of Science in Epidemiology in 1999. He continued his research activities at Children’s Hospital of Harvard Medical School until 2001.

In April 2007, he was appointed Attending Physician in the Infection Prevention and Control Service (SPCI) at HUG and also served as a consultant in the Infectious Diseases Service. He became head of the SPCI in October 2022.

A *Privat-docent* at the Faculty of Medicine since 2006, Professor Harbarth was appointed Associate Professor in 2010 and Full Professor in 2018. His research focuses on the epidemiology and prevention of antibiotic-resistant infections, as well as on antibiotic stewardship. A renowned expert in this field, he serves on numerous national and international expert committees. His work has received multiple awards, including the prestigious Robert Koch Award in 2022 for hospital hygiene and infection prevention.

His research group focuses on clinical and epidemiological studies aimed at addressing key issues related to the control of acquisition, transmission, and infection by multidrug-resistant organisms, as well as the associated clinical and public health burden. His work on the impact and control of nosocomial transmission of methicillin-resistant *Staphylococcus aureus* (MRSA) and extended-spectrum beta-lactamases (ESBL)-producing organisms has significantly improved strategies to combat these pathogens. His additional research interests include the molecular epidemiology of emerging bacteria, pharmaco-epidemiology and antibiotic optimization, and the rapid diagnosis of severe infections. He has been involved in several large-scale, EU-funded studies (REVERSE, ECRAID, COMBACTE) and led the major European project “Drive-AB,” which coordinated over 20 public and private partner institutions across 12 European countries to address this public health threat.

Professor Stephan Harbarth was included in the Highly Cited Researchers™ 2022 list.

## Your career has spanned clinical medicine, infectious diseases, and epidemiology. What initially drew you to the field of infection prevention and control, and how did your time at Harvard shape your research perspective?

I trained in Germany at a time when there was no formal specialty of infectious diseases. I started looking for places to train, which led me to Geneva in the mid-90s. At that time, everyone was focused on HIV and AIDS research, and one of the most prominent leaders in our field, Didier Pittet, had just returned from the United States. He was looking for a fellow, and surprisingly, no one was interested in joining him. I said, “OK.”

Because I was always interested in prevention and clinical research, I thought, why not join his group? That’s how I ended up in infection prevention and control (IPC). But to be honest, I didn’t wake up one morning during medical school thinking, “I have to go into infection control.” It was a natural evolution that led me to finding my “holy grail” in IPC.

Regarding the second part of your question—my time at Harvard in Boston was crucial. Those 3 years shaped my scientific agenda, strengthened my methodological skills, deepened my understanding of epidemiology, and broadened my scientific vision. I am very grateful to my mentors Don Goldman, Yehuda Carmeli, and Matt Samore, who helped guide my path. I also greatly enjoyed the stimulating intellectual environment there. It was a pivotal time for my career.

## You’ve held roles across multiple countries—Germany, Switzerland, and the United States. How have these international experiences influenced your approach to infection control and research, especially in collaboration?

I’ve been very fortunate to be exposed to a variety of healthcare settings, including electives in France, South Africa, Louisiana, and South Carolina—not only Massachusetts. This opened my eyes and gave me a broad, real-world perspective on healthcare issues beyond IPC and antimicrobial stewardship.

Once you spend time in different countries and healthcare systems, you see how much we can learn from one another. Cross-fertilization is essential. More than 25 years ago, I even published a comparison of antibiotic resistance and antibiotic use in the United States versus Germany—at that time, 2 very different cultural and healthcare contexts.

Let me share an anecdote: in 1992, during an elective in South Carolina, I was shocked to see a surgeon wearing surgical scrubs in a supermarket. That would have been unthinkable in Germany. These experiences highlight differences in culture, discipline, and practice.

Having an international background has also helped me coordinate large-scale European research networks. You must navigate different cultural expectations and working styles. My previous experiences helped me mitigate and mediate challenges—for example, during the pan-European project Drive-AB, which brought together European academic partners and US pharmaceutical companies and inevitably involved major conflicts. My background helped me manage those situations constructively.

## Your research focuses on the epidemiology and prevention of antibiotic-resistant infections. What do you consider the most significant recent breakthroughs in this field, and where do you see the greatest unmet challenges?

This is a very complex question, so I’ll break my response into several parts.

### Breakthroughs in mechanistic understanding

Recent observational studies have brought surprising findings. For example, MRSA may have emerged 150 years ago—well before antibiotics existed—and was part of the natural environment in hedgehogs. Understanding that some resistance is not purely man-made is an important insight.

For gram-negative “superbugs” like carbapenemase-producing Enterobacterales (CPE), major advances include understanding transmission pathways—especially plasmid-mediated transmission and the role of aquatic environments in hospitals. We’ve also gained insight into gut microbiota dynamics. Eric Pamer’s group in Chicago, for example, has produced outstanding work on how antibiotic exposure affects resistance determinants and how restoring the microbiota—eg, through fecal microbiota transplantation—can improve colonization resistance.

### Breakthroughs in IPC interventions

One of the major success stories is the global decline in hospital MRSA across many high-income countries. Although the exact drivers aren’t fully understood, I’m convinced that improved hand hygiene has played a large role.

Advances in diagnostics and sequencing have also helped us control resistance more effectively. From a clinical standpoint, reducing the duration of antibiotic treatment for many infections represents an important breakthrough that supports stewardship and reduces resistance risks.

### Unmet challenges

For IPC, a major unmet challenge remains individualized risk prediction. When a patient arrives at a hospital in France, Switzerland, the UK, or the United States, we still cannot reliably predict their risk for acquiring infections or resistant organisms.

Another ongoing challenge is behavioral change. Ensuring that healthcare workers consistently follow best practices will remain a struggle for as long as we practice medicine.

On the R&D side, we still lack promising vaccines—such as an effective *Staphylococcus aureus* vaccine. Furthermore, we need true innovation in antibiotic development; many new antibiotics remain closely related to preexisting ones. And from a public health perspective, we need far more progress in digitalization for surveillance, benchmarking, and monitoring antimicrobial resistance (AMR) and healthcare-associated infection rates.

## How do you see Europe’s role in shaping global antibiotic stewardship and infection prevention strategies?

Europe can play an important mediating role between the United States and Asia. We have a long tradition of hospital hygiene and infection control, with many thoughtful leaders, some of whom are not well known internationally but contribute exceptional ideas.

I believe Europe’s diversity can help shape future developments and bridge perspectives across continents. The European Centers for Disease Control and Prevention (ECDC) in Stockholm, for example, could take on a stronger role in certain international activities—not replacing the US CDC, but helping fill gaps. Unfortunately, the US CDC has been less visible internationally and less able to participate in major meetings in the past 12 months. We need strong voices in the IPC and AMR communities, and the ECDC could contribute significantly.

## You were instrumental in creating ICPIC, the International Conference on Prevention and Infection Control, held every 2 years in Geneva. What was the impetus for its creation, and what are the secrets to its ongoing success?

ICPIC began somewhat accidentally. First, there was a small think-tank meeting organized by a private diagnostic company. Didier Pittet, Andreas Voss, and I thought it was a shame to gather 30–40 world-class experts near Geneva without hosting a larger conference afterward. That was the initial idea—and it worked.

The first editions of ICPIC were essentially spin-offs of that think tank. Over time, we built the infrastructure for a full global meeting. The success likely came from the balance of practical and scientific sessions, the global scope, and strong participation from low- and middle-income countries. Clearly, there was an unmet need for a truly global IPC conference.

However, each edition remains a challenge. Without a major professional society behind us—like the Infectious Diseases Society of America (IDSA) or the European Society of Clinical Microbiology and Infectious Diseases (ESCMID)—it is always uncertain whether we will secure enough sponsors. During COVID, for example, we were close to running a deficit. So, each cycle is a risk, but so far, we’ve managed.

## How do you balance administrative leadership with active research?

It’s challenging, but I inherited an excellent team. Didier Pittet built a very strong structure, and we still have outstanding institutional support and resources here at HUG. This allows me to balance multiple responsibilities.

That said, my scientific productivity has declined somewhat because I can’t operate at full speed in every domain simultaneously. I’m also chairman of the clinical section of the medical school. Fortunately, I have excellent “first officers,” and it’s now my pleasure to mentor them and ensure they enjoy the same favorable conditions I had as a junior faculty member.

## Being named a Highly Cited Researcher in 2022 highlights the global impact of your work. What advice would you give early-career researchers aiming to produce research with real-world influence?

First: seek stimulating clinical research environments. Second: develop strong methodological skills—you must be able to conduct high-quality research. Third: don’t be afraid to challenge conventional wisdom. Fourth: go international and interdisciplinary. Step outside your comfort zone. Interact with colleagues you might not normally engage with. And finally, advice I often give junior colleagues:Be smart. Be humble. And always stay hungry. Always be willing to walk the extra mile.

## With leadership in mind, what qualities are essential for someone aiming to lead in infection control or clinical medicine?

You must be very diplomatic. A senior IPC leader in the United States once told me: “We are not first hospital epidemiologists. We are hospital diplomats.” I fully agree.

If you can be diplomatic listening to others, respecting their perspectives, you can build your entire leadership approach on that foundation. This was especially important during COVID. Instead of constantly correcting colleagues or acting like the “police,” I tried to understand their viewpoints and approach situations collegially. In French, we would say *bienveillant*—kind, respectful, constructive. That spirit is essential.

## Looking ahead, what emerging technologies or research areas hold the most promise for combating multidrug resistance and improving hospital hygiene?

AI and large language models will likely help us significantly over the next 10–20 years, particularly in personalizing infection prevention and control. Digitalization will also improve surveillance and benchmarking.

Sequencing will become cheaper and even more powerful, helping us track transmission and detect events earlier.

We must also make more progress in behavioral change—potentially through modern, technology-driven approaches.

Environmental reservoirs of resistant gram-negatives, especially sink and drain biofilms, remain a major challenge. Perhaps in 10 years we will have innovative technologies—including phage-based strategies—to better control these reservoirs. Similarly, other ideas are discussed in the “crystal ball” perspective we recently published in *Antimicrobial Resistance and Infection Control*.

## Tell us about the books on your nightstand. What do you like to read, and what inspires you?

I’m currently reading a novel by a young Swiss writer—about 23 years old—named Nielo Biedermann. The book is called *Lazar*. It hasn’t yet been translated but will be published in 25 languages. Some people say he may become the next Thomas Mann. It’s a beautifully written novel about several of his Hungarian family members over the past 100 years.

I’m also very interested in history. Humanity should never forget what happened 50, 100, or 1,000 years ago because human behavior doesn’t fundamentally change. I recently read a book by Julia Boyd and Angelika Patel titled *A Village in the Third Reich*. It is about a small town near where I grew up in Bavaria. The book explores how ordinary people managed daily life under the Nazi era dictatorship and war. Reading such histories gives me a lot to reflect on regarding current times: how we behave, whether we are opportunists or heroes, and how we respond to challenges today.

